# Haspin Modulates the G2/M Transition Delay in Response to Polarization Failures in Budding Yeast

**DOI:** 10.3389/fcell.2020.625717

**Published:** 2021-01-28

**Authors:** Martina Galli, Laura Diani, Roberto Quadri, Alessandro Nespoli, Elena Galati, Davide Panigada, Paolo Plevani, Marco Muzi-Falconi

**Affiliations:** Dipartimento di Bioscienze, Università degli Studi di Milano, Milano, Italy

**Keywords:** polarization, mitosis, actin cytoskeleton, cell cycle, *Saccharomyces cerevisiae*, morphogenesis checkpoint

## Abstract

Symmetry breaking by cellular polarization is an exquisite requirement for the cell-cycle of *Saccharomyces cerevisiae* cells, as it allows bud emergence and growth. This process is based on the formation of polarity clusters at the incipient bud site, first, and the bud tip later in the cell-cycle, that overall promote bud emission and growth. Given the extreme relevance of this process, a surveillance mechanism, known as the morphogenesis checkpoint, has evolved to coordinate the formation of the bud and cell cycle progression, delaying mitosis in the presence of morphogenetic problems. The atypical protein kinase haspin is responsible for histone H3-T3 phosphorylation and, in yeast, for resolution of polarity clusters in mitosis. Here, we report a novel role for haspin in the regulation of the morphogenesis checkpoint in response to polarity insults. Particularly, we show that cells lacking the haspin ortholog Alk1 fail to achieve sustained checkpoint activation and enter mitosis even in the absence of a bud. In *alk1*Δ cells, we report a reduced phosphorylation of Cdc28-Y19, which stems from a premature activation of the Mih1 phosphatase. Overall, the data presented in this work define yeast haspin as a novel regulator of the morphogenesis checkpoint in *Saccharomyces cerevisiae*, where it monitors polarity establishment and it couples bud emergence to the G2/M cell cycle transition.

## Introduction

The yeast *Saccharomyces cerevisiae* reproduces through a budding process in which the daughter cell growth is promoted prior to anaphase, thus defining the orientation of the future mitotic spindle. This process starts in G1, when a cluster of proteins collectively known as the polarisome is built up at the presumptive bud site to drive symmetry breaking from an otherwise round cell. A major player in this polarization step is the small GTPase Cdc42, which oversees every step of the polarization process ranging from actin organization, to septin deposition and vesicle delivery (Etienne-Manneville, 2004). Cdc42 is regulated by an intricate mechanism to timely promote polarity onset and bud emergence and later in the cell-cycle polarity dispersion and cytokinesis. The main determinants of Cdc42 activation are the essential GEF Cdc24, whose differential localization directs when and where polarity clusters are established (Zheng et al., 1994; Caviston et al., 2002), and its GDI (Rdi1) and GAPs (Rga1, Rga2, Bem2, and Bem3) (Pierce and Clark, 1981; Marquitz et al., 2002; Smith et al., 2002; Tiedje et al., 2008). In particular, localized recruitment and activity of Cdc24 is essential to promote symmetry breaking and the consequent bud emergence. Given the absolute requirement for a bud to the cell-cycle of budding yeast, it is not surprising that a surveillance mechanism, known as the morphogenesis checkpoint exists to delay mitotic progression in presence of polarization insults that impair bud emergence and growth (Lew and Reed, 1995a; McMillan et al., 1998). This pathway acts through an inhibitory phosphorylation of Cdc28 on Y19, which is catalyzed by the kinase Swe1 (Gould and Nurse, 1989; Harvey et al., 2005) (Wee1 in higher eukaryotes) and reverted by the phosphatase Mih1 (Russell and Nurse, 1986, 1987; Dunphy and Kumagai, 1991; Gautier et al., 1991) (Cdc25). In case of altered polarization, and thus impaired budding, the morphogenesis checkpoint provides the cells the chance to achieve an efficient polarity establishment and bud emergence before entering mitosis. Once a proper cellular morphogenesis is restored, Swe1 is degraded and Mih1 removes Cdc28-Y19 phosphorylation allowing completion of the cell cycle (Sia, 1998; McMillan et al., 2002; Kellogg, 2003; Asano et al., 2005; McNulty and Lew, 2005; Raspelli et al., 2011; Anastasia et al., 2012; King et al., 2013). In contrast with this wt scenario, mutants defective for the morphogenesis checkpoint undergo mitosis even in the presence of non-polarized, unbudded cells; resulting in nuclear division within a single cell compartment (Russell et al., 1989; Booher et al., 1993; Lew and Reed, 1995a; Sia et al., 1996; McMillan et al., 1998; Harvey and Kellogg, 2003; Keaton and Lew, 2006). Most works have focused on the ability of the morphogenesis checkpoint to inhibit mitotic entry. However, activation of this process was also found to cause delays later during mitosis, primarily in metaphase, through inhibition of APC/C activity (Barral et al., 1999; Sreenivasan and Kellogg, 1999; Theesfeld et al., 1999; Carroll et al., 2005; Chiroli et al., 2007; Lianga et al., 2013). A further complication comes from evidence in budding yeast showing that the deletion of *MIH1* induces only mild delays in mitotic entry and anaphase onset, suggesting the possible contribution of other phosphatases (Russell et al., 1989; Rudner et al., 2000; Pal et al., 2008; Lianga et al., 2013). This hypothesis was confirmed by the discovery that Mih1, Ptp1, and PP2A^Rts1^ act redundantly to regulate the spatial and temporal reactivation of Cdc28, collaborating to its stepwise triggering prior to anaphase onset (Kennedy et al., 2016). Swe1 and Mih1 are temporally and spatially modulated by various factors. The regulatory circuits monitoring their activity involve Hsl1, Hsl7, Cla4, and Cdc5, which promote Swe1 phosphorylation at the septin ring (Barral et al., 1999; Longtine et al., 2000; Crutchley et al., 2009). Hyper-phosphorylated Swe1 is ubiquitinated by the Met30/SCF complex, which targets it for Cdc34-dependent proteolysis (Kaiser et al., 1998). Mih1, on the other hand, undergoes dramatic changes in phosphorylation throughout the cell cycle in a Cdc28 and casein kinase 1-dependent manner (Pal et al., 2008). Though the contribution of Mih1 phosphorylation to its activity is still debated, there are reports showing that during the G2/M transition Mih1 is dephosphorylated and activated by Cdc55-dependent PP2A phosphatase (Carroll et al., 2005; Wicky et al., 2011).

Haspin is an atypical serine/threonine atypical kinase that phosphorylates H3-T3 during metaphase, promoting the recruitment of the chromosomal passenger complex (CPC) at kinetochores (Tanaka et al., 1999; Higgins, 2001a,b; Kelly et al., 2010; Wang et al., 2010). Accordingly, depletion of haspin in mammalian cells prevents proper chromosome positioning at the metaphase plate, eventually blocking cell-cycle progression in mitosis (Dai and Higgins, 2005; Dai et al., 2005, 2006; Yamagishi et al., 2010). Haspin activity is cell-cycle dependent, with the protein being held in an inactive state during interphase through folding of an autoinhibitory domain onto the catalytic one (Kelly et al., 2010). This autoinhibition is relieved during mitosis by Cyclin-dependent kinase 1 (CDK1)-mediated phosphorylation at haspin N-terminus, followed by further phosphorylations at multiple sites by the Polo-like kinase 1 (Plk1). These Plk1-dependent modifications trigger haspin activity, resulting in phosphorylation of H3-T3 (Ghenoiu et al., 2013; Zhou et al., 2014). The genome of *Saccharomyces cerevisiae* encodes for two haspin paralogues, Alk1 and Alk2 (Nespoli et al., 2006), whose levels peak in mitosis and G2-phase, respectively, and that are phosphorylated during the cell cycle (Spellman et al., 1998; Nespoli et al., 2006). We previously reported that Alk1 and Alk2 are critical to efficiently disperse polarity clusters in mitosis (Quadri et al., 2020a), preventing cell death in case of transient mitotic delays (Panigada et al., 2013). In agreement with Alk1 and Alk2 being, at least partly, redundant, these phenotypes have been observed in double deleted strains, with single mutants behaving as their wt counterparts.

Here we report that budding yeast haspin homolog Alk1 exerts an independent function, playing a critical role in the regulation of the G2/M transition in response to morphogenetic stress. Cells deleted for *ALK1* are indeed defective in the morphogenesis checkpoint and are characterized by a premature Cdc28-Y19 dephosphorylation. Intriguingly, the phenotypes of *alk1*Δ mutants are suppressed by concomitant deletion of *ALK2*. Accordingly, we show evidence for a precocious and higher inhibition of Cdc28 in *alk2*Δ strains, supporting a role for Alk2 in quenching of the morphogenesis checkpoint.

## Results

### Haspin Regulates Cell-Cycle Progression Upon Defective Polarization

**Figure 1:**
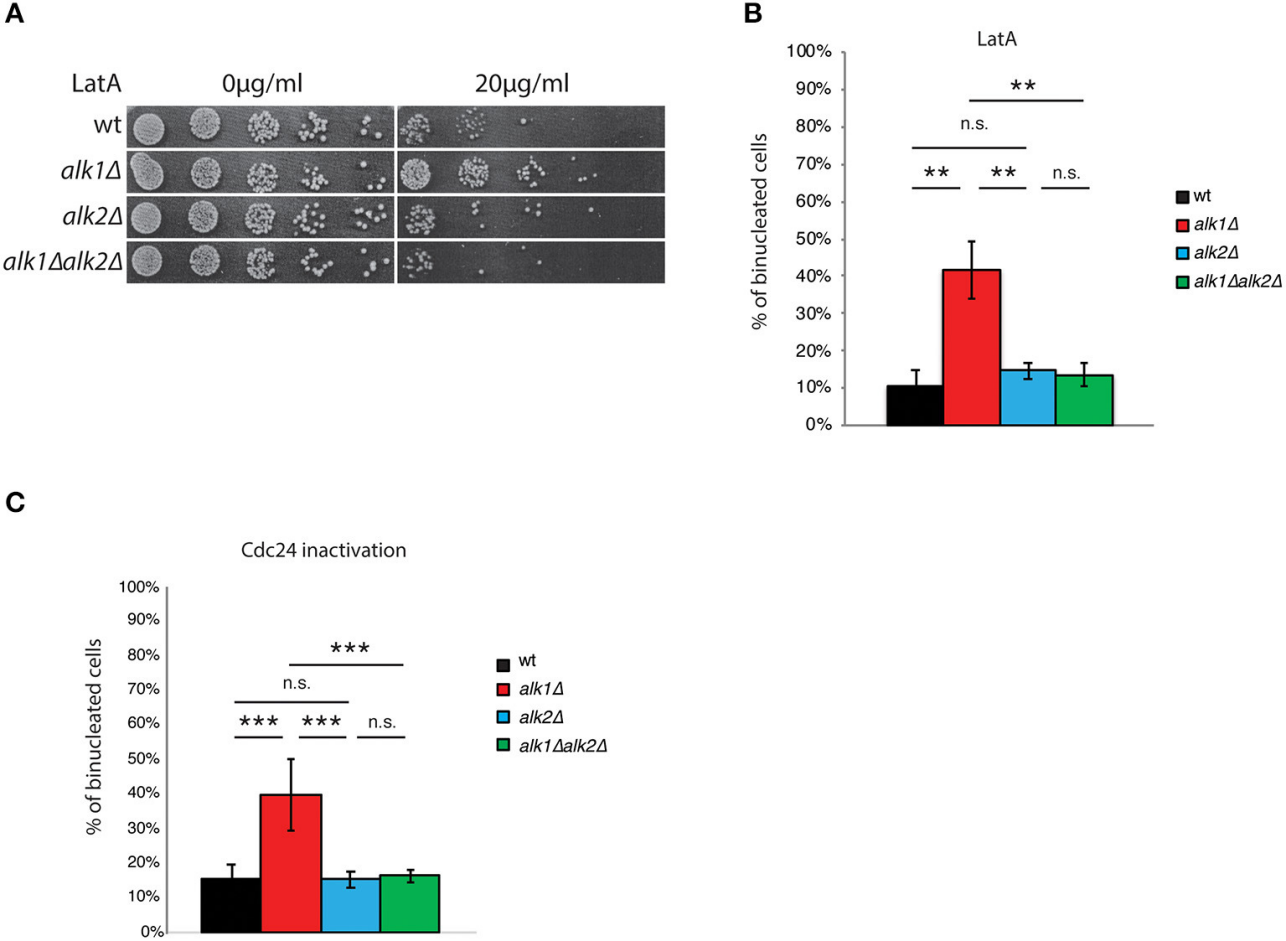
Haspin mutants show altered response to polarity failures. **(A)** Serial dilutions of cultures of the indicated strains were spotted on either DMSO or LatA containing plates. After 24 h incubation at 28°C plates were imaged. **(B)** Cells of the indicated strains expressing Tub1-GFP were arrested in G1 with mating pheromone and then released in the presence of 100 μM LatA. After 4 h cells were fixed and analyzed for nuclear division by fluorescence microscopy. **(C)** Cells were arrested in G1 at the permissive temperature (25°C), shifted for 45' at the restrictive temperature (37°C) and then released at 37°C. After 2 h, samples were fixed and analyzed for nuclear division, as above. Error bars in **(B,C)** represent standard deviation, statistical significance was measured by *T*-test, ns: not significant, **p < 0.01, ***p < 0.005.

The atypical protein kinase haspin has been shown to be involved in the promotion of a proper alignment of the chromosomes on the metaphase plate (Dai and Higgins, 2005; Dai et al., 2005; Kelly et al., 2010; Wang et al., 2010, 2011; Yamagishi et al., 2010) and in cell polarity (Panigada et al., 2013; Quadri et al., 2020a,b). To expand our comprehension of haspin and identify other possible functions, we tested the sensitivity of haspin-lacking cells to a set of non-genotoxic compounds. Interestingly, we found that the deletion of *ALK1*, but not of *ALK2*, suppressed the sensitivity of yeast cells to the actin depolymerizing drug LatA (Figure 1A). Such suppression required the activity of Alk2, indeed the concomitant deletion of *ALK2* restored the LatA sensitivity of *alk1*Δ mutants to that of control strains (Figure 1A).

LatrunculinA is a powerful natural toxin that, by binding actin monomers, prevents their polymerization (Spector et al., 1983; Ayscough et al., 1997). In budding yeast, a deficient organization of the actin cytoskeleton impairs, among other processes bud emission. Cells thus arrest of cell-cycle progression as single cells with and undivided nucleus through activation of the morphogenesis checkpoint (Lew, 2003). To better characterize the influence of *ALK1* on the cellular response to LatA treatment, we analyzed nuclear division following exposure to LatA in control cells or cells lacking Alk1, Alk2, or both. Tub1-GFP expressing cells were synchronized in G1 with mating pheromone and then released into fresh medium containing 100 μM LatA. Nuclear dynamics was monitored 4 h after the release, scoring the percentage of cells with two nuclei and the presence of anaphase spindles. As shown in Figure 1B; Supplementary Figures 1A,B, LatA treatment prevents anaphase in wt strains, where a marginal fraction (11%) of cells becomes binucleated in these conditions. In agreement with the phenotype observed by drop assays, *alk1*Δ cells exhibited a reduced response to LatA treatment, as seen by nuclear division (42% binucleated cells) and spindle elongation (29% anaphase spindles). Deletion of *ALK2*, which does not significantly alter the cellular sensitivity to LatA of control strains, rescues the defects observed in *alk1*Δ cells.

To test whether the observed phenotypes were specific to LatA treatment or a common feature of haspin mutants upon G1 polarization defects, we employed a genetic approach to interfere with bud emission. Cdc24 is the main guanine nucleotide exchange factor (GEF) for Cdc42, the master regulator of cellular polarity in budding yeast (Adams et al., 1990; Zheng et al., 1994; Bi et al., 2000). Among the plethora of processes directly regulated by Cdc42 are polarity establishment, actin dynamics and bud emergence, and hence impaired Cdc42 activity during G1 ultimately leads to the activation of the morphogenesis checkpoint as a consequence of polarity impairments (Miller and Johnson, 1997). We thus exploited a *cdc24-1* mutant that upon shift to restrictive temperature is unable to sustain polarization, thus triggering the morphogenesis checkpoint (Sloat et al., 1981).

First, we verified whether, as observed upon LatA treatment, loss of *ALK1* improved the fitness of *cdc24-1* strains upon polarization defects. To this end, we tested the growth of *cdc24-1* and *cdc24-1alk1*Δ strains at permissive (25°C), semi-permissive (32°C) or restrictive (37°C) temperatures. As shown in Supplementary Figure 1C, loss of *ALK1* promoted the growth of cells with impaired Cdc24 activity at 32°C, confirming an improved fitness of cells lacking Alk1 in presence of polarization insults. We then monitored Alk1 contribution to nuclear segregation upon chronic exposure to Cdc24 inactivation. Wild-type, *alk1*Δ*, alk*Δ, and *alk1*Δ *alk2*Δ cells in *cdc24-1* background were grown at 25°C (permissive temperature), arrested in G1 with α-factor and shifted at 37°C (non-permissive temperature) for the last 45 min of the treatment, in order to deplete Cdc24 activity before budding onset. Cells were then released from the G1 arrest into fresh medium at 37°C to promote cell cycle progression in presence of budding defects. The kinetics of nuclear division was analyzed by fluorescence microscopy. As shown in Figure 1C, cells expressing wild-type haspin delay anaphase onset so that only a small fraction (15%) of the population underwent nuclear division at 120' when budding is defective due to mutated *CDC24*. Consistently with what observed with LatA, in *cdc24-1alk1*Δ strains the mitotic delay is defective and binucleated cells reach 40% by 2 h after the G1 release. This phenotype is again suppressed by concomitant loss of Alk2.

Up to now, most of the roles played by haspin are exerted through phosphorylation of H3-T3. Thus, we verified whether the phenotypes observed upon loss of Alk1 could be ascribed to altered histone phosphorylation. To this end, we incubated wt, *H3-T3A* and *alk1*Δ*H3-T3A* strains with 100μM Lat A for 4 h and then calculated the percentage of binucleated non-budded cells. As shown in Supplementary Figure 1D, loss of histone phosphorylation *per se* does not lead to unscheduled nuclear division in these conditions, suggesting that, whatever the role played by haspin in this pathway, it is not dependent on H3-T3 phosphorylation.

Overall, these observations indicate that yeast *ALK1* plays a role in the cellular response to polarization insults in the early stages of the cell cycle. Surprisingly, this function is not shared between haspin paralogues. Indeed, our results suggest a role for Alk1 in promoting of the morphogenesis checkpoint, while Alk2 seems to have an opposite role.

### Alk1 Regulates Cell Cycle Progression Through Mih1 Inactivation

**Figure 2:**
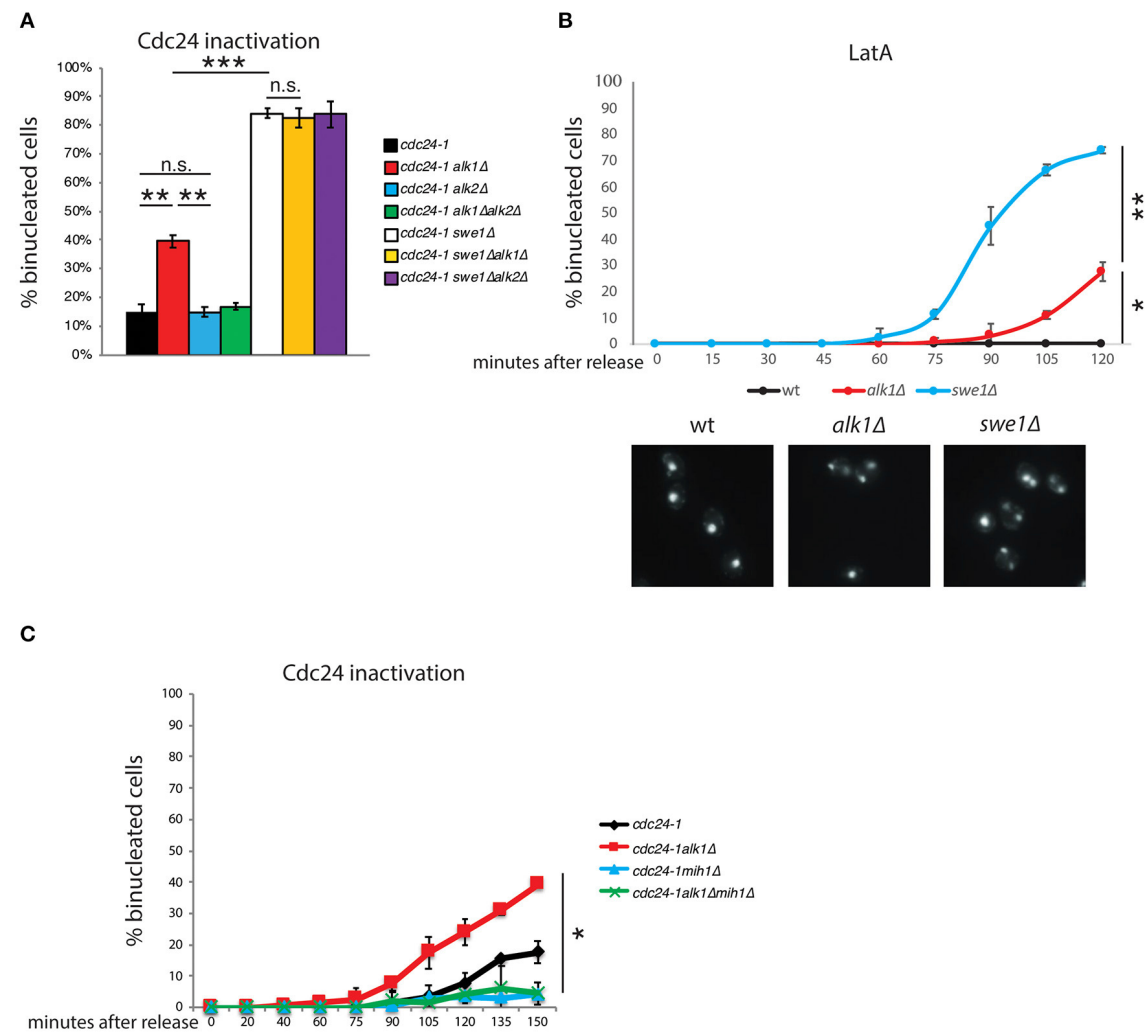
Alk1 regulates cell cycle progression through Mih1. Cells of the indicated strains were arrested in G1 at 25°C and held at 37°C for further 45' before being released at 37°C. After 2 h samples were taken and nuclear segregation was evaluated by fluorescence microscopy **(A)** For wt, *alk1*Δ, *alk2*Δ and *alk1*Δ*alk2*Δ, the mean value was calculated on data from experiment in Figure 1C combined with new biological replicates performed together with the other indicated strains. **(B)** Cells of the indicated strains were synchronized in G1 and treated with LatA. Following release, samples were taken to monitor nuclear division by fluorescence microscopy. Representative images at the 120' time point are shown **(C)** Cells were treated as in A, taking samples at the indicated time points to monitor nuclear segregation or cell-cycle progression (Supplementary Figure 2). Error bars represent standard deviation, statistical significance was measured by *T*-test, ns: not significant, *p < 0.05, **p < 0.01, ***p < 0.005.

In budding yeast, budding impairments trigger a surveillance mechanism, known as the morphogenesis checkpoint, which delays mitotic entry (Lew and Reed, 1995a; McMillan et al., 1998). Swe1 kinase phosphorylates Cdc28-Y19 (Gould and Nurse, 1989; Harvey et al., 2005), inhibiting its function and preventing entry into mitosis. The phosphatase Mih1 is largely responsible for the removal of the phosphate group, releasing the cell cycle arrest (Sia et al., 1996; Harvey and Kellogg, 2003). The premature resumption of cell cycle progression observed in *alk1*Δ cells suggests that Alk1 may positively modulate Swe1 or act as an inhibitor of Mih1, preventing the G2/M transition in the presence of polarity problems. Similarly, Alk2 could act on Swe1 or Mih1 with an opposite role. To obtain clearer insights on the possible interplay between haspin and Swe1 in the control of Cdc28 activity we analyzed different mutants in a *cdc24-1* background, following the release from a G1 arrest at the restrictive temperature. We then assessed how loss of *ALK1* or *ALK2* affected the nuclear segregation of cells lacking *SWE1*. As shown in Figure 2A, loss of Alk1 or Alk2 does not further worsen or ameliorate the defects of *swe1*Δ mutants, suggesting that haspin is indeed involved in the regulation of the morphogenesis checkpoint (see Supplementary Figure 2A for cell-cycle analysis). We then compared the kinetics of nuclear division in *ALK1* and *SWE1* mutants following LatA treatment in G1 (Figure 2B). Interestingly, we found that, besides being defective compared to control cells, nuclear segregation in *alk1*Δ strains is delayed compared to that of cells completely lacking Swe1 kinase, suggesting defects in sustained morphogenesis checkpoint activity. If the morphogenetic insult is prolonged, in the absence of both Alk1 or Swe1, cells fail to arrest and undergo multiple rounds of DNA replication as nuclear division even in the absence of a bud, leading to the formation of polynucleated cells (Supplementary Figure 2B).

In the presence of polarity insults that trigger the morphogenesis checkpoint, Mih1 is expected to be inactive (Harrison et al., 2001; Ciliberto et al., 2003). Thus, two possibilities can explain the observed defects in Alk1 mutants: loss of Alk1 either causes a failure in sustaining Swe1 activity or it promotes unscheduled Mih1 activation. If the loss of *ALK1* results in the unscheduled activation of Mih1, deletion of *MIH1* should suppress the phenotypes of Alk1-lacking cells. On the other hand, if *ALK1* deletion causes defective Swe1 activity, the concomitant loss of Mih1 would not impact *alk1*Δ phenotypes since Mih1 should be inactive in these conditions. As shown in Figure 2C (see Supplementary Figure 2C for cell-cycle analysis), while loss of Alk1 led to an anticipated nuclear division, additional deletion of *MIH1* restored normal anaphase kinetics, confirming the epistatic relation between Alk1 and Mih1. Noteworthy, *MIH1* deletion alone do not affect anaphasic nuclei at 90'-105'-120' after the release, when *alk1*Δ strains already exhibit nuclear segregation, confirming that the phosphatase is inactive in control cells. This observation further supports the proposed unscheduled activation of Mih1 upon loss of Alk1.

**Figure 3:**
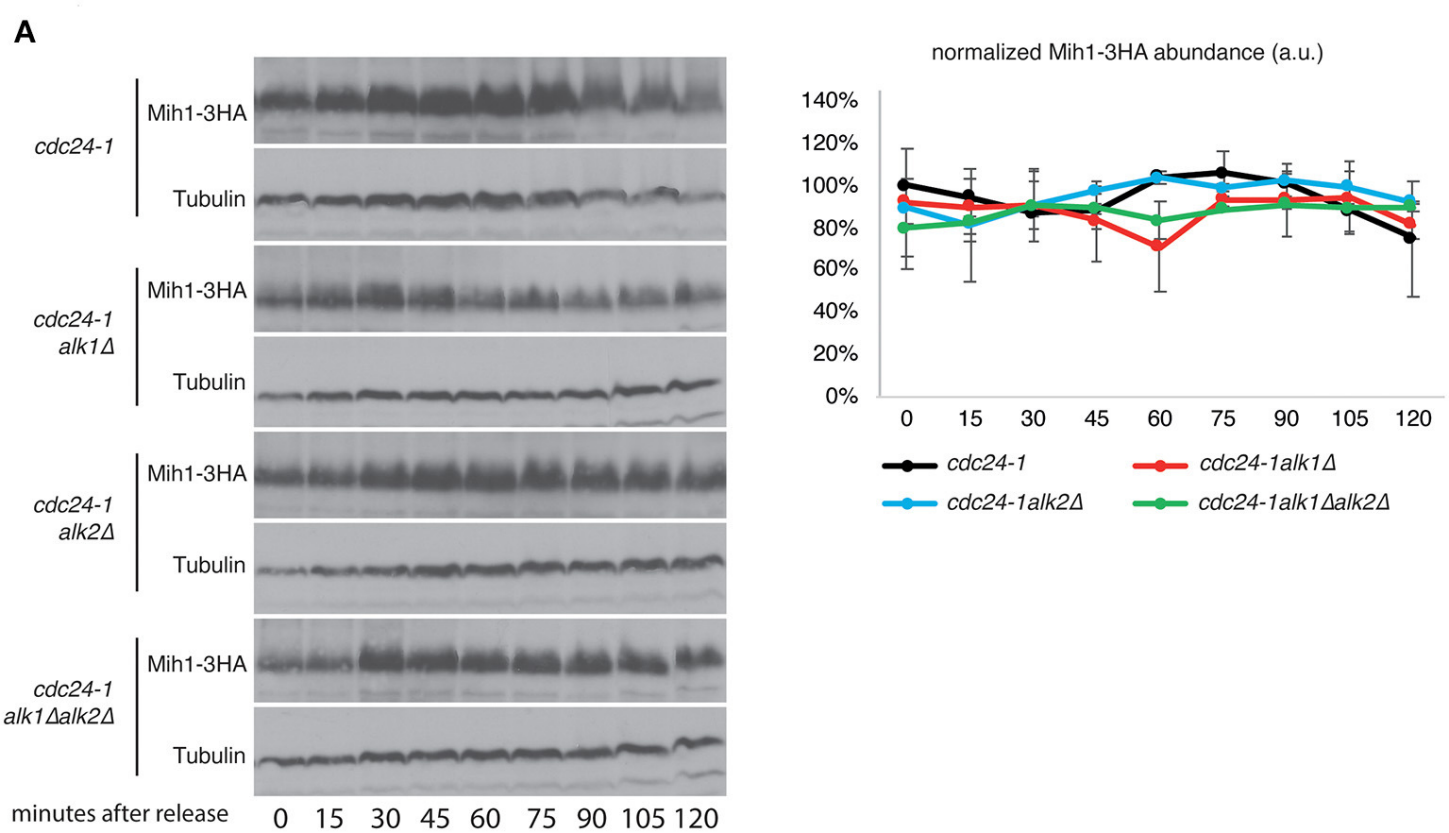
Alk1 does not modulate Mih1 post-translational modifications. **(A)** Cells of the indicated strains were synchronized in G1 at permissive temperature and held at 37°C for further 45' before being released into the cell cycle at restrictive temperature. Samples were taken every 15' to follow protein levels and modifications by western blot. The graph shows the relative Mih1-3HA abundance normalized on tubulin levels. Error bars represent standard deviation.

This regulation is unlikely to be direct. Indeed, no physical interaction between Alk1 and Mih1 (nor Swe1) was detected by two-hybrid (Supplementary Figure 3A; a strain expressing LexA-p53 and B42-3HA-SV40 was used as a positive control). The morphogenesis checkpoint main regulators, Mih1 and Swe1 are both tightly controlled in a posttranslational manner which involves several kinases and phosphorylation events (Pal et al., 2008). We then hypothesized that Alk1 and Alk2 could exert their role in this pathway by regulating Mih1 phosphorylations. However, we did not observe significant differences in both Mih1 and Swe1 protein levels or posttranslational modifications in haspin mutants (Figure 3A; Supplementary Figure 3B).

### Alk1 and Alk2 Modulate Cdc28-Y19 Phosphorylation Upon Defective Budding

**Figure 4:**
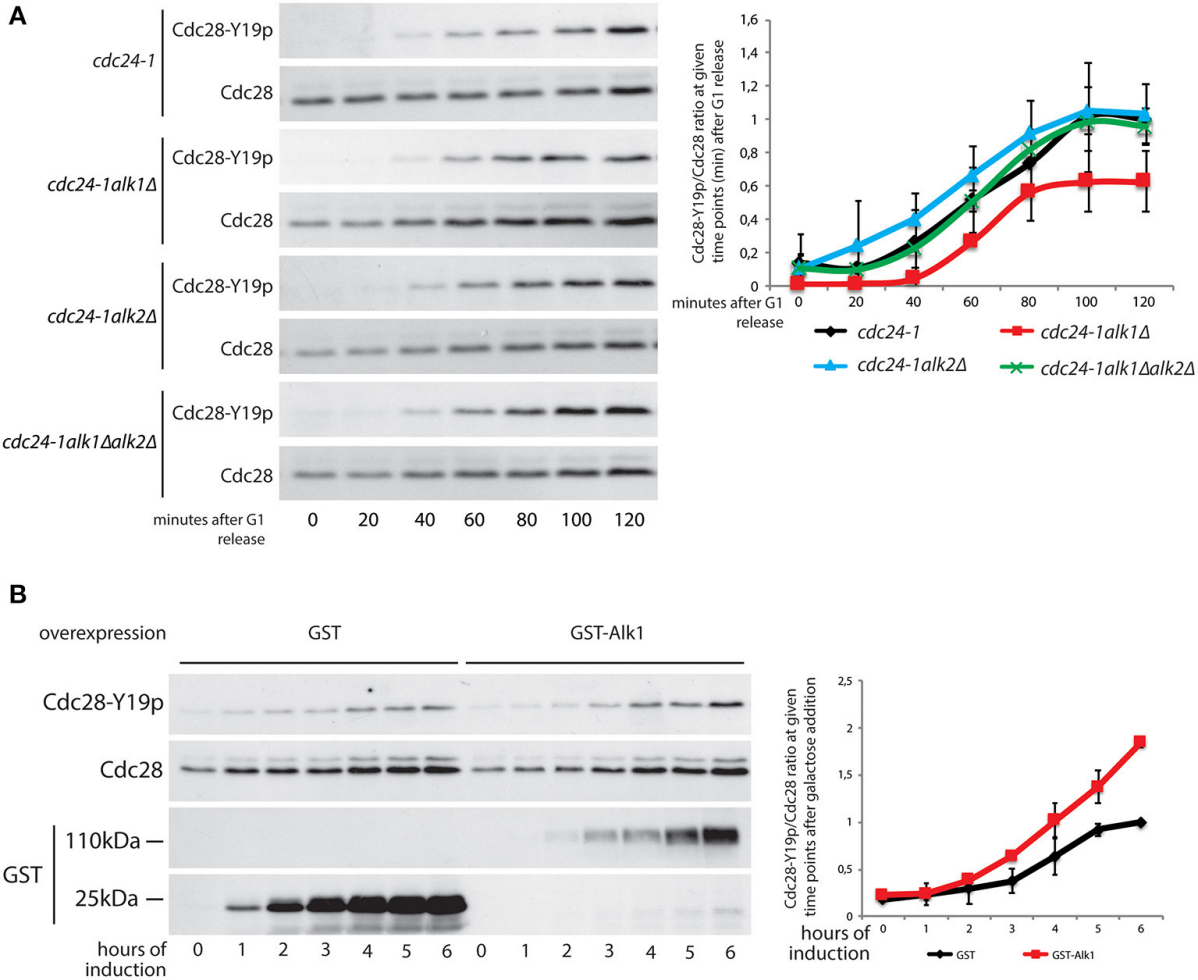
Alk1 is required for sustained Cdc28-Y19 phosphorylation. **(A)** Cells of the indicated strains were synchronized in G1 at permissive temperature and held at 37°C for further 45' before being released into the cell cycle at restrictive temperature. Samples were taken every 20' to follow protein levels by western blot using antibodies for total Cdc28 and phosphospecific antibodies for Cdc28-Y19p. The right panel reports the quantification of the phosphorylation signals normalized over the total Cdc28. Error bars represent standard deviation **(B)** Logarithmically growing cells, bearing the pGAL-*GST* or pGAL-*GST-ALK1* constructs, were incubated in the presence of 2% galactose to induce protein overexpression. Samples were taken every hour to monitor protein levels. Western blotting was performed with antibodies for total Cdc28 and phosphospecific antibodies for Cdc28-Y19p. Expression levels of GST and GST-Alk1 were analyzed with anti GST antibodies. The graph shows the ratio between phosphorylated Cdc28-Y19 and total Cdc28, error bars represent standard deviation.

Previous results show an interplay between yeast haspin and the morphogenesis checkpoint in case of unpolarized cells. To directly address haspin involvement in the activation and maintenance of this checkpoint we monitored the impact of haspin loss on the kinetics of Cdc28-Y19 phosphorylation in synchronized cultures. *cdc24-1, cdc24-1alk1*Δ, *cdc24-1alk2*Δ, and *cdc24-1alk1*Δ*alk2*Δ strains were arrested in G1, shifted to non-permissive temperature to deplete Cdc24 activity and released in pheromone-free medium, taking samples at different time points. The levels of phosphorylated Cdcd28-Y19 were measured with phosphospecific antibodies and fluorescence-based analysis. As shown in Figure 4A, (see Supplementary Figure 4A for cell cycle analysis) loss of Alk1 does not impede Cdc28-Y19 phosphorylation, but it prevents its accumulation after ~1 h from the release. Again, removal of Alk2 restores a wt inactivation of Cdc28 in *alk1*Δ cells. To reinforce the notion that Alk1 is a positive regulator of the morphogenesis checkpoint, we exploited a reversed approach, where we overexpressed the kinase and monitored the accumulation of Cdc28-Y19p. As shown in Figure 4B; Supplementary Figure 4B, increased *ALK1* levels indeed caused elevated levels of phosphorylated Cdc28-Y19 with no evident effect on cell-cycle progression, further supporting our conclusions.

## Discussion

A proper timing between different developmental events is fundamental for successful cell-cycle completion and proliferation of every organism. In *Saccharomyces cerevisiae*, cell division occurs by budding and this requires that a daughter cell is formed prior to anaphase. It is the mother-bud axis, pre-defined in G1 by setting up polarity clusters, that will determine the direction of spindle elongation. In this scenario, failures in symmetry breaking and cellular polarization impede bud emission and lead to a cell-cycle arrest with replicated DNA in a single nucleus (Lew and Reed, 1995b). The molecular mechanism that couples nuclear dynamics and budding is known as morphogenesis checkpoint, a network able to perceive defects in bud formation and to transduce this stimulus in an inhibitory phosphorylation of CDK1 (Cdc28-Y19p) (Sia et al., 1996). Two proteins act as master regulators of the morphogenesis checkpoint, the kinase Swe1 and the phosphatase Mih1 (WEE1 and CDC25 in higher eukaryotes, respectively); the concerted activity of these players directly regulate Cdc28-Y19 phosphorylation (Booher et al., 1993; Sia et al., 1996).

The atypical kinase haspin targets H3-T3 and has been ascribed with several roles in nuclear dynamics, ranging from chromosome cohesion to chromatin condensation, alignment at the metaphase plate and asymmetric histone inheritance (Dai and Higgins, 2005; Dai et al., 2005, 2006; Kelly et al., 2010; Wang et al., 2010, 2011; Yamagishi et al., 2010; Tran et al., 2012; Ghenoiu et al., 2013). Here we report an unprecedented involvement of budding yeast haspin paralogues Alk1 and Alk2 in the morphogenesis checkpoint. In particular Alk1 seems to play a positive role in delaying the cell cycle progression upon failures in polarity establishment and bud emergence. Indeed, cells lacking *ALK1* exhibit an abortive cell cycle arrest in response to the actin cytoskeleton poison LatA and following genetic inactivation of the polarity regulator Cdc24. This last observation excludes that the LatA sensitivity phenotype could be due to a reduced cell wall permeability of *alk1*Δ cells or to LatA specific effects. Alk2 has an opposite role and its loss is sufficient to restore normal phenotypes in Alk1 mutants. This failure in cell-cycle arrest is due to a defective inhibitory phosphorylation of Cdc28-Y19 in *alk1*Δ strains, which is again suppressed by concomitant loss of Alk2. In particular, *alk1*Δ cells are able to generate an initial, increase in levels of phosphorylated Cdc28-Y19, which however fails to accumulate to the levels of wt strains. This could be explained both by a loss in Swe1 functionality or by an unscheduled Mih1 activation.

However, deletion of *MIH1* completely suppresses the defects due to loss of *ALK1*. Notably, this suppression occurs at a stage when in wt cells Mih1 itself is inactive, clearly identifying Mih1 and not Swe1 as the branch regulated by Alk1. Our two hybrid results suggest that Alk1 does not directly interact with Mih1 or Swe1, and we found no evident contribution in terms of posttranslational modifications of Mih1 or Swe1 by Alk1.

All the proteins analyzed here are conserved in human cells, where Wee1 (Swe1) and Cdc25 (Mih1) have crucial activities in the control of cell cycle, and their misfunction is often coupled with carcinogenesis. The mechanism by which *WEE1* and *CDC25* become deregulated during cancer development remains still unclear. Conceptually, we therefore believe that understanding haspin contribution to the Wee1/Cdc25 pathway can shed light in long term on mechanisms underlying tumor development. The mechanism linking haspin to the morphogenesis checkpoint is still elusive. Both Swe1 and Mih1 are subjected to extensive phosphorylation, and it has been technically very challenging to link haspin activity to it. The involvement of H3-T3 phosphorylation is unlikely as this PTM is restricted to mitosis, while the morphogenesis checkpoint arrests cells at the G2/M transition. This suggest that other still unidentified haspin targets may be relevant to this pathway.

## Methods

### Yeast Strains and Plasmids

Yeast strains used in this study are isogenic to W303 apart from those used in two-hybrid assays, marked with ^*^, which are isogenic to EGY48, and those marked with ^**^, which are isogenic to UCC1111, and are listed in Table 1. Conditions for yeast cell cultures used have been previously described (Rose et al., 1990). When indicated the cultures were synchronized in G1 by 2 μg/ml α-factor as previously described (Foiani et al., 1994). Standard molecular genetics techniques were used to construct plasmids and strains. In particular, PCR-based genotyping were used to confirm gene disruption and tagging (Longtine et al., 1998).

**Table 1:** Strains and plasmids used in this work.

**Name**	**Relevant Genotype**	**Source**
**STRAINS**		
K699	*ade2-1, trp1-1, can1-100, leu2-3, his3-11,15, ura3 MATa*	K.Nasmyth
EGY42	*ura3 his3 trp1 6xLexAop-LEU2 MATa*	R.Brent
UCC1111	*adh4::URA3-TEL (VII-L) hhf1-hht1::LEU2 hhf2-hht2::MET15 [HHF2-HHT2]* MATα	Parthun's Lab (Kelly et al., 2000)
SP1791	*TUB1-GFP::HIS3 MATa*	Piatti's Lab
YPD294	*TUB1-GFP::HIS3 alk1::NATr MATa*	This work
YPD414/1A	*TUB1-GFP:HIS3 alk2::KANr MATa*	This work
YPD298	*TUB1-GFP::HIS3 alk1::NATr alk2::KANr MATa*	This work
YPD274	*cdc24-1 MATa*	This work
YPD280/9A	*cdc24-1 alk1::NATr MATa*	This work
YPD282/12A	*cdc24-1 alk2::KANr MATa*	This work
YPD282/5A	*cdc24-1 alk1::NATr alk2::KANr MATa*	This work
**YPD226	*UCC1111 [HHT2-T3A]* MATα	This work
**YRQ549	*UCC1111 alk1::KANr [HHT2-T3A]* MATα	This work
YPD458	*cdc24-1 swe1::LEU2 MATa*	This work
YPD459	*cdc24-1 alk1::NATr swe1::LEU2 MATa*	This work
YPD460	*cdc24-1 alk2::KANr swe1::LEU2 MATa*	This work
Q225	*swe1::LEU2 MATa*	This work
YPD286/10C	*cdc24-1 mih1::TRP1 MATa*	This work
YPD288/7A	*cdc24-1 alk1::NATr mih1::TRP1 MATa*	This work
*YLD123	*[pSH18-34] [B42-3HA] [LexA-ALK1] MATa*	This work
*YLD125	*[pSH18-34] [B42-3HA-MIH1] [LexA] MATa*	This work
*YLD124	*[pSH18-34] [B42-3HA-SWE1] [LexA] MATa*	This work
*YLD127	*[pSH18-34] [B42-3HA-MIH1] [LexA-ALK1] MATa*	This work
*YLD126	*[pSH18-34] [B42-3HA-SWE1] [LexA-ALK1] MATa*	This work
*YMIC1D7	*[pSH18-34] [B42-3HA-SV40] [LexA-p53] MATa*	Lab stock
YLD18/20C	*cdc24-1 MIH1-HA-TRP1 MATa*	This work
YLD19/13A	*cdc24-1 alk1::KANr MIH1-HA-TRP1 MATa*	This work
YLD20/3D	*cdc24-1 alk2::HIS3 MIH1-HA-TRP1 MATa*	This work
YLD21/10D	*cdc24-1 alk1::KANr alk2::HIS3 MIH1-HA-TRP1 MATa*	This work
YPD336/6A	*cdc24-1 SWE1-HA-URA3 MATa*	This work
YPD338/11A	*cdc24-1 alk1::KANr SWE1-HA-URA3 MATa*	This work
YPD339/9C	*cdc24-1 alk2::HIS3 SWE1-HA-URA3 MATa*	This work
YPD341/7C	*cdc24-1 alk1::KANr alk2::HIS3 SWE1-HA-URA3 MATa*	This work
YAN64-2	*[pGAL1-GST]*	This work
YAN78-1	*[pGAL1-GST-ALK1]*	This work
**PLASMIDS**		
pPD9	*PMP3-HHT2-T3A*	This work
pSH18-34	*8xLexAop-LacZ*	R.Brent
pJG4-5	*pGAL1-B42AD-HA*	R.Brent
pEG202	*pADH-LexA*	R.Brent
pAN5	*pEG202-ALK1*	This work
pLD22	*pJG4-5-SWE1*	This work
pLD23	*pJG4-5-MIH1*	This work
p53	*pEG202-p53*	Lab stock
TAg	*pJG4-5-SV40TAg*	Lab stock
pEG(KT)	*pGAL-GST*	Lab stock
pAN8	*pGAL-GST-ALK1*	This work

### Latrunculin a Treatment

Cells were grown in YPD medium, synchronized in G1 with α-factor (2 μg/ml) and released in the presence of LatA (SIGMA L5163) 100 μM for 240 min. Cells were then harvested for protein extraction or fixed for microscopy analysis.

### Spindle Elongation and Nuclear Division Analysis

Cells carrying *TUB1-GFP* were fixed with formaldehyde (3.7%) and washed three times with PBS. GFP was visualized by fluorescence microscopy with a Leica DMRA2 widefield fluorescence microscope equipped with a CCD camera (Leica DC 300F). For the analysis of nuclear division cells were fixed with ethanol, washed three times in PBS and DNA was stained with DAPI. Labeled-DNA was visualized by fluorescence microscopy as described above. Images were processed by ImageJ (Schindelin et al., 2012). Nuclear division pattern was evaluated by scoring for unbudded cells showing a single nucleus or two nuclei. At least 300 cells were categorized per sample across three experimental repeats to calculate a mean and a standard deviation.

### Morphogenesis Checkpoint Assays

To evaluate morphogenesis checkpoint activation cells carrying *cdc24-1* temperature-sensitive allele were grown at 25°C (permissive temperature), arrested in G1 with α-factor (2 μg/ml), shifted for 45 min at 37°C (non-permissive temperature) and released at 37°C. At indicated time points, samples were collected, fixed in ethanol and stained with DAPI. Nuclear division was evaluated as described above. Trichloroacetic acid protein extraction was used to evaluate Cdc28-Y19 phosphorylation by Western blot. The ratio between Cdc28-Y19 phosphorylation and total Cdc28 was performed on protein levels of three independent experiments.

### Western Blot

To analyze proteins during kinetic experiments samples were collected at given time points and exposed to trichloroacetic acid precipitation (Muzi Falconi et al., 1993). Protein extracts were then resolved by SDS- PAGE and analyzed by Western blot using proper antibodies. Anti-HA antibodies (12CA5) were used as previously described (Sabbioneda et al., 2007). Anti-phospho-Cdc2 (Tyr15) (#9111, Cell Signaling), anti-Cdc2 (ab17) (#ab18-100, Abcam) and anti-GST (#27-4577-01V, GE Healthcare) were used with standard techniques. Images were taken with a ChemidocTouch Imaging System (Bio-Rad) and processed with ImageLab and ImageJ.

### Two-Hybrid

EGY42 cells were transformed with the indicated plasmids (pEG202, pJG4-5 and their derivatives expressing fusions with Alk1, Mih1, or Swe1). Fusion proteins were checked by western blots. The *lacZ* reporter is harbored on the pSH18-34 plasmid. Relevant strains were patched on selective raffinose/galactose-containing plates supplemented with 0.195 nM X-Gal, 23.1mM NaH_2_PO_4_ and 21.1mM Na_2_HPO_4_. Pictures were taken after overnight incubation at 28°C.

### Cell Cycle Analysis With FACScan

Samples were taken at given time points, fixed with ethanol and processed with RNase A and Proteinase K. Cells were then stained with 1 μM SytoxGreen and DNA content was determined using a FACScan cytofluorimeter.

## Data Availability Statement

The raw data supporting the conclusions of this article will be made available by the authors, without undue reservation.

## Author Contributions

MG, LD, RQ, MM-F, and PP planned the experimental approach, revised the experiments and analyzed the data. MG, RQ, and MM-F wrote the manuscript. MG, LD, RQ, AN, and DP performed the experiments. EG and DP contributed to experimental procedures and discussion. All authors contributed to the article and approved the submitted version.

## Conflict of Interest

The authors declare that the research was conducted in the absence of any commercial or financial relationships that could be construed as a potential conflict of interest.
