# Supplementary figures and images for: Haspin Modulates the G2/M Transition Delay in Response to Polarization Failures in Budding Yeast
Source: Front Cell Dev Biol. 2021 Jan 28;8:625717. doi: 10.3389/fcell.2020.625717 (PMC7876276; doi:10.3389/fcell.2020.625717)

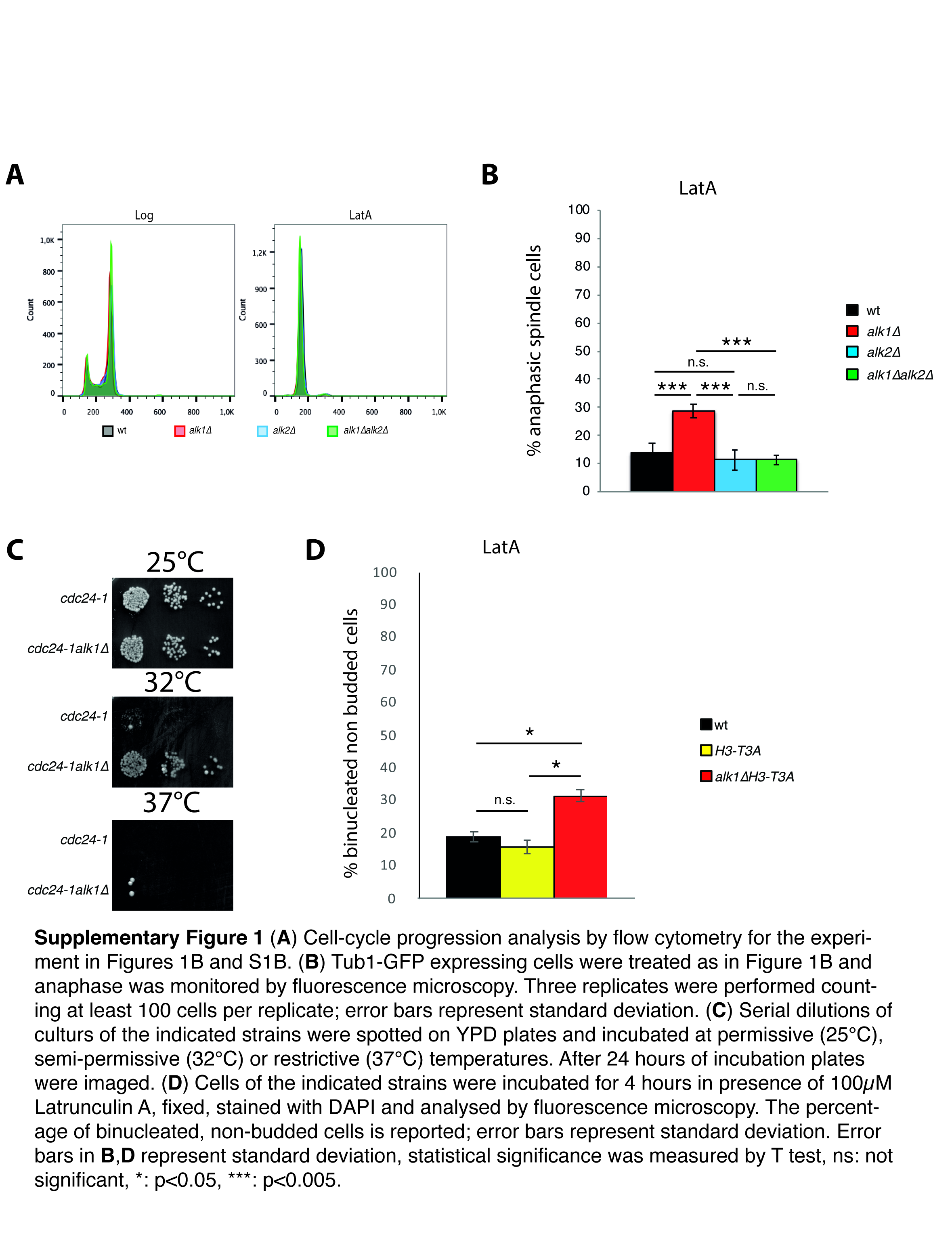

Supplement: Supplementary file 1 [file Image_1.tif]

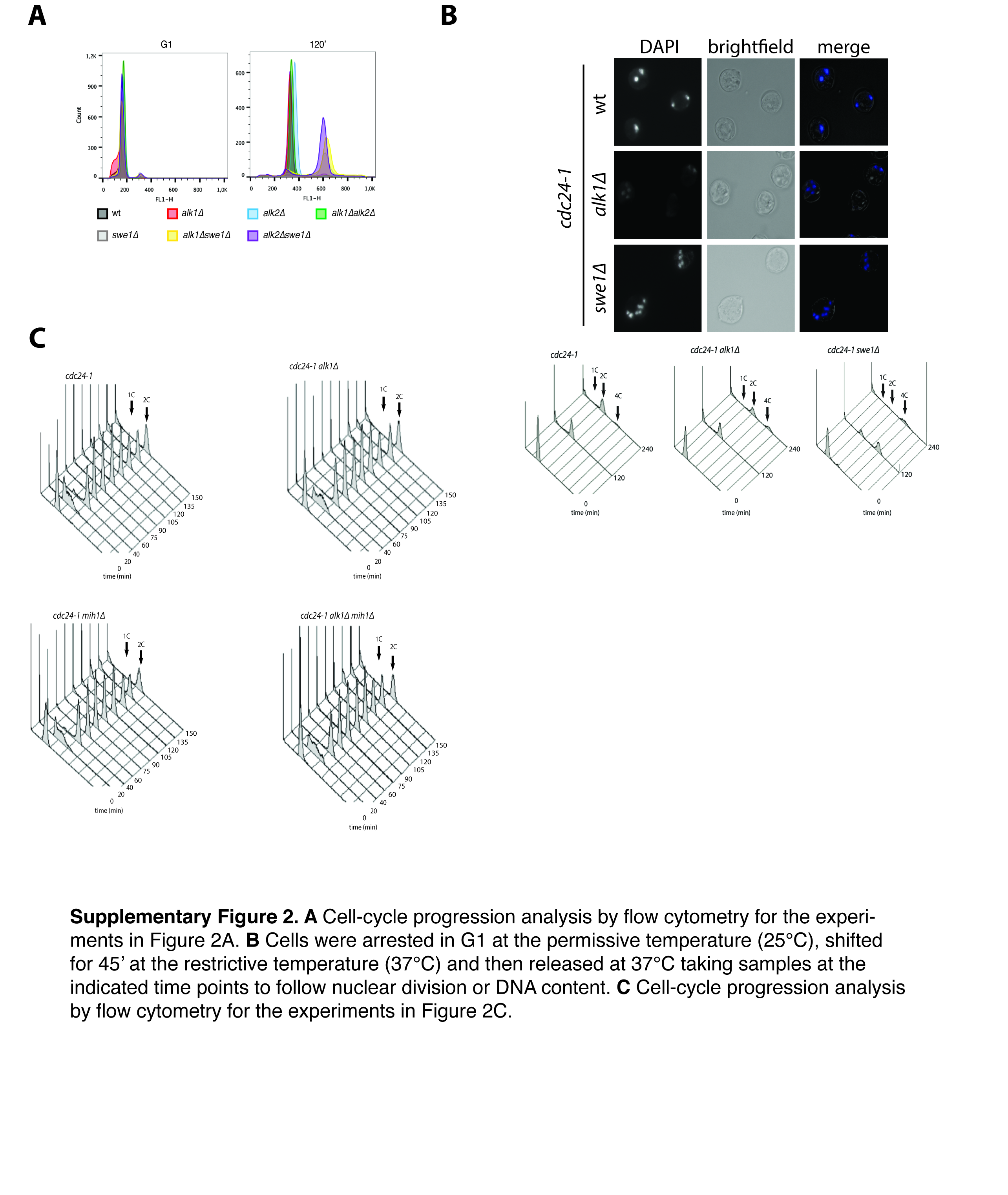

Supplement: Supplementary file 2 [file Image_2.TIF]

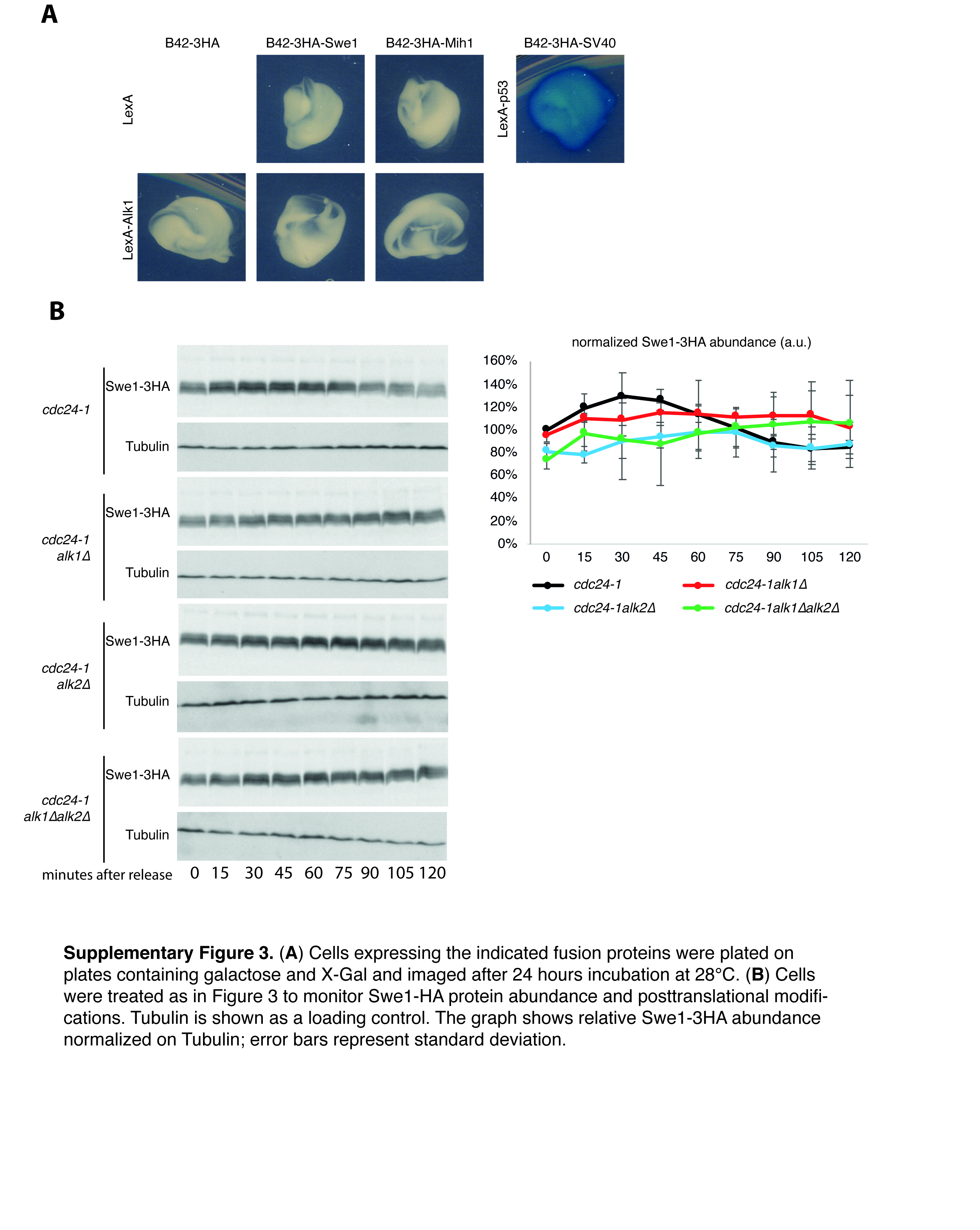

Supplement: Supplementary file 3 [file Image_3.TIF]

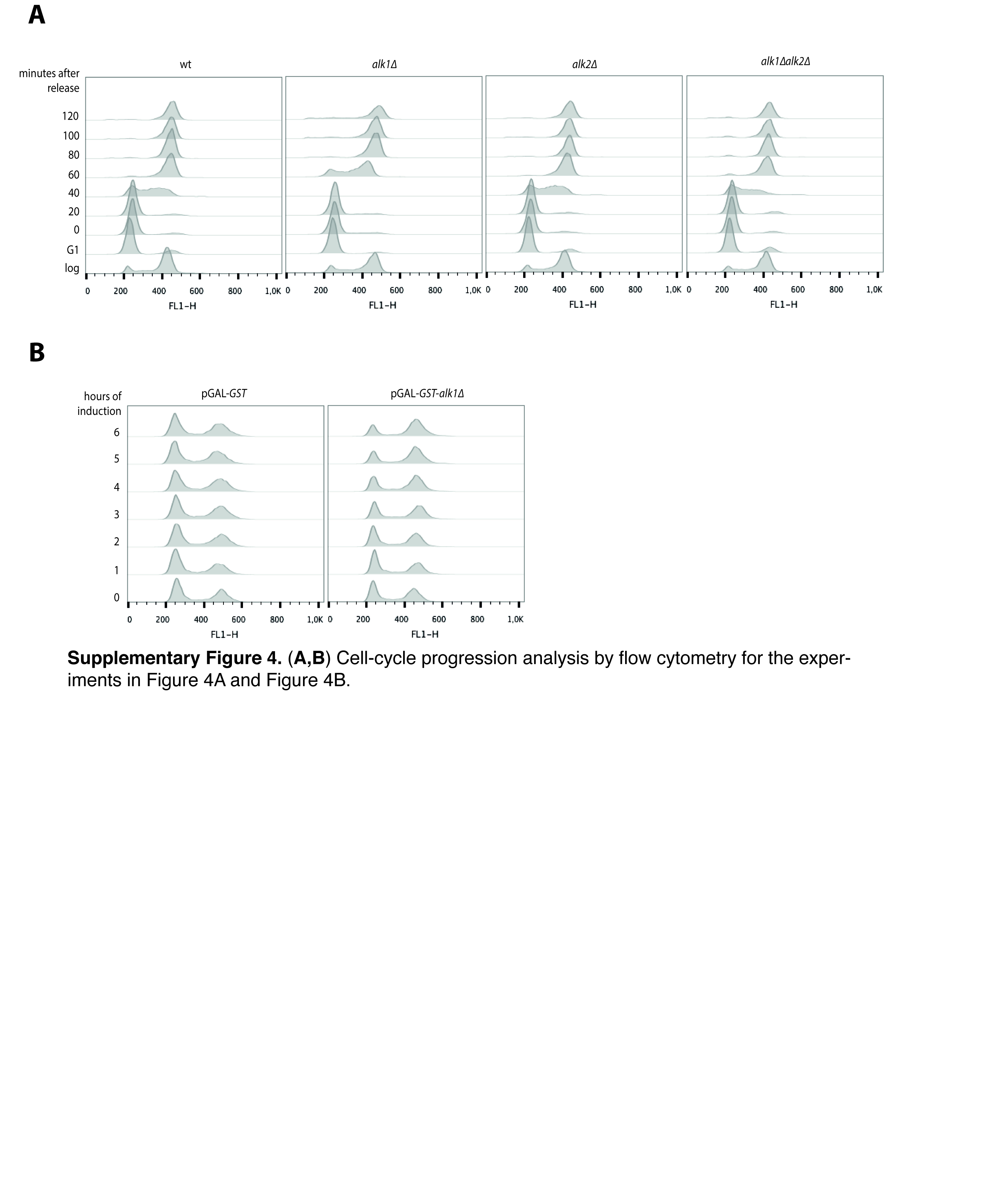

Supplement: Supplementary file 4 [file Image_4.TIF]
